# The Relationship Between Periodontal Disease and ABO Blood Groups: A Cross-Sectional Study

**DOI:** 10.3290/j.ohpd.b1452963

**Published:** 2021-06-01

**Authors:** Mansour Al-Askar, Hani S. AlMoharib, Razan Alaqeely, Arwa A. Talakey, Hamad Alzoman, Abdulmonem Alshihri

**Affiliations:** a Associate Professor and Periodontist, Department of Periodontics and Community Dentistry, College of Dentistry, King Saud University, Riyadh, Saudi Arabia. Study design, patient examination, data collection, wrote manuscript.; b Assistant Professor and Periodontist, Department of Periodontics and Community Dentistry, College of Dentistry, King Saud University, Riyadh, Saudi Arabia. Patient examination, edited and reviewed manuscript.; c Lecturer, Department of Periodontics and Community Dentistry, College of Dentistry, King Saud University, Riyadh, Saudi Arabia. Study design review, collected data.; d Dental Public Health Postgraduate Student and Demonstrator, Department of Periodontics and Community Dentistry, College of Dentistry, King Saud University, Riyadh, Saudi Arabia. Study design review, patient examination, collected data.; e Associate Professor and Periodontist, Department of Periodontics and Community Dentistry, College of Dentistry, King Saud University, Riyadh, Saudi Arabia. Methodology design, edited and reviewed manuscript.; f Associate Professor and Prosthodontist, Department of Prosthetic Dental Science, College of Dentistry, King Saud University, Riyadh, Saudi Arabia. Statistical analysis, edited and reviewed manuscript.

**Keywords:** ABO blood-group system, genetic, gingivitis, periodontitis, Rh-Hr blood-group system

## Abstract

**Purpose:**

The objective of this cross-sectional study was to evaluate the relationship between ABO blood groups and periodontal diseases.

**Materials and Methods:**

Four hundred sixteen subjects (223 females, 193 males) were recruited according to the eligibility criteria. Periodontal examination was performed, including full-mouth plaque index (PI), bleeding on probing (BOP), clinical attachment level (CAL), and interproximal bone loss (IBL). ABO blood group patterns were determined based on self-reports, confirmed by medical records. The chi-squared test was done to evaluate the data (p < 0.05).

**Results:**

Out of the 416 subjects, 52.2% were blood group O, whereas 27.8% were blood group A. 46.8% of patients with blood group O had gingivitis and 49.6% had periodontitis. 31.2% of patients with blood group A had gingivitis,while 29.5% had periodontitis. The blood group with the lowest percentage among patients with gingivitis was AB, with a rate of 6.2%; in this blood group, 8.1% had periodontitis.

**Conclusions:**

There is no association between periodontal diseases and ABO blood group types.

Periodontal diseases affect the gingival tissue and alveolar bone.^19^ Aetiopathogenesis is based on several factors. The presence of bacterial biofilm and the defensive immunity response constitute the primary aetiological factors for periodontal disease initiation.^1^ Moreover, the clinical manifestation of periodontal disease may differ according to the pathological bacterial composition, the individual’s somatic homeostasis, and many other local and systemic factors.^17^ The host’s defense mechanism may be influenced by various systemic factors such as smoking, diabetes, and genetic disposition.^21^ The aetiology of periodontal disease is still an area meriting exploration to identify markers that can determine disease initiation risks.^8^ An innate factor is the putative correlation between periodontal disease and blood groups.^2^

Ever since Karl Landsteiner discovered ABO blood groups in 1900,^25^ several systemic health conditions have been found to be associated with blood group differences.^23^ According to the antigens present on red blood cells, individuals were classified as possessing agglutinogen A, agglutinogen B, both A and B (AB), or neither (O).^25^ It is interesting to note that these antigens are present in body fluids and tissue fluids in hapten format, called the ‘group-specific substances’.^23^ Moreover, the distribution of ABO blood groups varies globally.^2,10^

Oral diseases have been evaluated in terms of the ABO groups to determine the absence or presence of an association. A limited number of studies were found in the literature that explored the relationship between ABO groups and periodontal diseases.^10^ Nonetheless, outcomes were controversial and based on the type of disease and geographic differences. A correlation study was conducted in 1971 by Kaslick et al^12^ in the US. Seven patients who were impacted by aggressive periodontitis were included in the study.^12^ It was found that blood group B was more strongly correlated with aggressive periodontitis than was the case among blood-group O patients. Two other studies explored the connection with aggressive periodontitis, finding either AB or B groups in 20 affected Nigerian patients, while either O or AB groups were identified in 15 affected Indian patients.^3,18^ An evident association has not been proven; however, the correlation remains a possibility. Many studies have been conducted on the relationship between ABO and chronic periodontitis. In 1971, Pradhan et al^23^ conducted the first study; however, it failed to demonstrate any association. Three recent studies showed that group O was dominant in patients affected by chronic periodontitis.^16,18,27^ Pai et al^22^ revealed a blood-group O dominance in gingivitis patients, whereas group B was most frequent in patients with chronic periodontitis. These outcomes resembled those of Gautam et al,^10^ conducted on 537 patients. The study by Demir et al,^9^ which included 1351 subjects, revealed a high prevalence of chronic periodontitis in patients with blood group O.

Greater insight into the genetic variation and its link with periodontal disease and severity might be required for early identification, prevention, and care. This research was conducted to study the link of gingivitis and periodontitis to ABO blood groups and Rh factor in patients with periodontal diseases. The the null hypothesis was that there is no association between periodontal diseases and ABO blood types.

## Materials and Methods

The present study was a single-center, cross-sectional clinical observational study conducted from February to December 2019.

### Ethical Considerations

Ethical approval was obtained from the Institutional Review Board (E-18-0284) of King Saud University, Riyadh, Saudi Arabia. The study was performed in accordance with the Declaration of Helsinki of 1975, as revised in 2013. Informed consent was obtained from all participants.

### Study Population and Study Design

The study was performed on patients referred to the periodontics clinic. The subjects who met the following criteria were included in this clinical study: ages 18–65 years; systemically healthy; having at least 20 teeth, not including the third molars. The exclusion criteria were: had periodontal treatment in the last three months; antibiotic use in the previous six months; smokers; pregnancy. Periodontal diseases were classified according to the 2017 World Workshop on the Classification of Periodontal and Peri-Implant Diseases and Conditions.^26^ Periodontitis was defined as interdental clinical attachment loss (CAL) noticeable at ≥ 2 non-adjacent teeth, oral or buccal CAL ≥3 mm with pocketing > 3 mm at ≥ 2 teeth. Gingivitis was defined as periodontal pocket depth of less than 3 mm, with no radiographic loss of bone, but displayed signs of inflammation, such as color, contour, gingival bleeding, change in surface texture, e.g. oedema.^20^

Periodontal clinical measurements were performed by two trained and calibrated periodontists blinded to participants’ case/control status. Probing depth (PD) was recorded at six points around all teeth using a periodontal probe; measurements were rounded to the lowest whole millimeter^6^ (Williams probe, Hu-Friedy; Chicago, IL, USA). In addition, clinical attachment level (CAL), bleeding on probing (BoP) and interproximal bone loss (IBL) were recorded. During the examination, radiographs included digital complete-mouth serial radiographs, bitewings, and/or a panoramic radiograph. Duplicate examinations in 10% of the participants (one random quadrant) were conducted to assess intra- and inter-examiner reliability. The intra-examiner reliability values (intraclass correlation) were 0.87 (PD) and 0.91 (CAL) for dentist 1 and 0.89 (PD) and 0.94 (CAL) for dentist 2. The inter-examiner reliability values were 0.89 and 0.93 for PD and CAL, respectively. Subjects were asked to report their ABO blood group based on their medical records.

### Statistical Analysis

A sample size calculation was performed using nQuery advisor (Version 7.0, Statistical Solutions; Cork, Ireland) to achieve a type 1 rate of α=0.05 and power of 85%. Statistical analysis was performed using SPSS version 26 (SPSS; Chicago, IL, USA). The chi-squared test was used to determine statistically significant differences between the study groups. p-values ≤ 0.05 were defined as statistically significant.

## Results

### Demography of the Participants

The current study was undertaken on 416 subjects, of whom 223 (53.6%) were females and 193 (46.4%) were males. The age ranged from 20-65 years, with the mean age 39.4 ± 10.7 years. In accordance with the periodontal diagnosis, the subjects were split into two different groups. There were 188 patients with gingivitis in group 1, with 98 females and 90 males, whereas group 2 consisted of 228 patients with periodontitis (125 females and 103 males).

### ABO Blood Group Distribution

**Fig 1:**
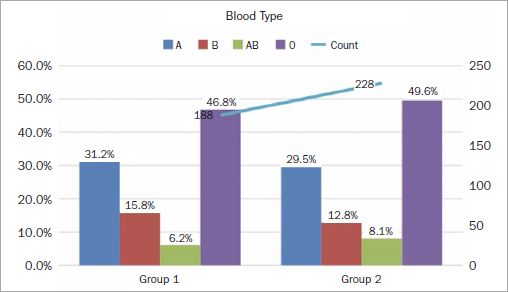
Survey results by blood type.

Out of the 416 subjects examined, 52.2% were blood group O, whereas 27.8% were blood group A. 10.8% were Rh-negative and 89.2% were Rh-positive. Figure 1 displays ABO blood groups’ frequency distribution in general and in patients with periodontitis and gingivitis. A high frequency of periodontal disease was found in subjects with blood groups O and A. A statistically significantly higher percentage of patients with blood group O had gingivitis (46.8%), while 49.6% had periodontitis.

The second most frequent blood group among gingivitis patients was type A (31.2%), while periodontitis was found in 29.5% of this group. The least frequent blood group in gingivitis patients was AB with 6.2%; 8.1% of periodontitis patienst had the blood group. In this study, the blood group distribution in both groups was not statistically significantly different (p>0.05).

### Rh Factor Distribution

**Fig 2:**
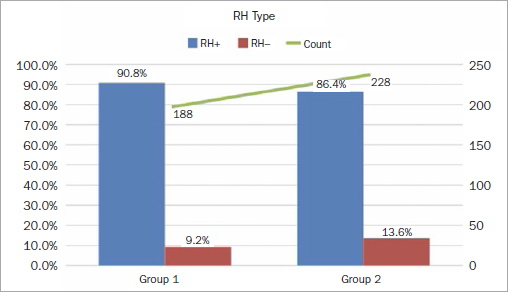
Survey results by Rh type.

The Rh-positive genotype was more frequent than the Rh-negative. However, this applies to every ABO genotype. Rh-negative was found in nearly 10.8% of every genotype, and nearly 89.2% were Rh-positive (shown in Fig 2). The current study compared the Rh-factor distribution among two study groups. However, no statistically significant differences were seen concerning the distribution of the Rh-factors between the two groups.

## Discussion

Periodontal diseases are serious infections which may result in tooth loss if left untreated.^7^ Bacterial bioflim is the primary cause of periodontal disease. Nevertheless, several background factors, including gender, education, age, oral hygiene routines, genetic characteristics, smoking habits, and socioeconomic status have been recognised as risk factors for periodontal diseases.^8,17,21^

ABO blood-type proportions vary among populations according to race.^15^ This is also the case for periodontal diseases, the distribution of which can differ between races.^14^ Considering this aspect, the question arises if the percentage of ABO blood subgroup distribution works has a relevant impact on the rate of periodontal diseases in different societies. Little evidence is available on the incidence of dental and oral ailments in relation to ABO blood groups.

A study by Arowojolu et al^3^ reported a difference in the association between hemoglobin types B and AB and juvenile and non-juvenile periodontitis. A few authors have reported that certain ABO blood groups were highly associated with a risk of oral diseases^11,23^ – for instance, group O was more associated with periodontitis – ^23^ while another study found no association.^13^ The studies mentioned above provided preliminary data about the relationship between periodontal diseases and ABO blood groups, as stated in Al-Askar’s review article.^2^

The present study explored the link between periodontal diseases and ABO blood groups. We found that a high frequency of blood group O is seen in patients with gingivitis and periodontitis, representing the overall sample of the study. Nevertheless, Gawrzewska et al^11^ identified blood-group O patients as having a high severity of periodontal diseases, whereas blood-group A patients had a higher resistance towards periodontal diseases. On the other hand, Pradhan et al^23^ revealed substantial alterations when ABO blood groups were linked with different groups of periodontal severity included in their study.The results of the current study are in line with those of Barros and Witkop,^4^ in which there were no statistically significant differences between subjects with or without periodontal disease regarding ABO blood groups. Furthermore, in this study, blood group O was more dominant in general, agreeing with data published from Saudi Arabia.^5,24^ Concerning the Rh-factor, no statistically significant difference in the occurrence of periodontitis was found between the Rh+ and Rh- when compared with the gingivitis group.

A major limitation of the present study is that clear data interpretation was not possible, as a reference group comprising periodontally healthy individuals was not assessed. It is important to mention that gingivitis is merely a transient stage which, if not treated in a timely manner, results in loss of supporting alveolar bone (periodontitis). The authors suggest that further studies including a reference group of periodontally healthy patients (BOP < 10%, PPD ≤ 3 mm and non-CAL) should be performed to evaluate the association between the ABO blood groups and periodontitis.

## Conclusion

There is no association between periodontal diseases and ABO blood types. These results should be cautiously interpreted, as the ABO blood groups were not assessed in periodontally healthy individuals. Nevertheless, long-term follow-up studies with varying geographic distribution and inclusion of periodontally healthy individuals are required to make a detailed assessment of the influence of ABO blood groups on periodontal diseases.
